# Development of interleukin-17-producing Vγ2^+^ γδ T cells is reduced by ICOS signaling in the thymus

**DOI:** 10.18632/oncotarget.8464

**Published:** 2016-03-29

**Authors:** Terkild Brink Buus, Jonas Damgård Schmidt, Charlotte Menné Bonefeld, Carsten Geisler, Jens Peter Holst Lauritsen

**Affiliations:** ^1^ Department of Immunology and Microbiology, Faculty of Health and Medical Sciences, University of Copenhagen, Copenhagen, Denmark

**Keywords:** γδ T cell, development, thymus, ICOS, interleukin-17, Immunology and Microbiology Section, Immune response, Immunity

## Abstract

Co-stimulation is an integral part of T cell signaling involved in almost all facets of T cell biology. While much is known about co-stimulation in differentiation and function of conventional αβ T cells, less is known about how co-stimulation affects the development and programming of γδ T cells. In this study, we have investigated the role of inducible T cell co-stimulator (ICOS) on the development of γδ T cells. We show that ICOS is expressed by a population of immature Vγ2^+^CD45RB^low^ γδ T cells predisposed to interleukin-17 (IL-17) production. We found that treatment with ICOS specific antibodies drastically reduces fetal development of IL-17-producing γδ T cells by agonistic actions, and that ICOS deficient mice have a significant increase in the population of IL-17-producing Vγ2^+^ γδ T cells in the thymus, spleen, lymph nodes and skin and exhibit exacerbated sensitization responses to 2,4-dinitrofluorobenzene. In conclusion, this study demonstrates that development of IL-17-producing Vγ2^+^ γδ T cells is reduced by ICOS signaling in the thymus.

## INTRODUCTION

T cells are a critical part of the adaptive immune system and can be divided into two distinct lineages based on their expression of either the αβ or the γδ T cell receptor (TCR). αβ T cells recognize peptide antigens presented on classical MHC molecules. Antigen recognition leads to clonal expansion of the antigen-specific αβ T cells and mounting of a highly specific albeit delayed adaptive immune response. In contrast, γδ T cells can recognize more diverse antigens and mount a rapid response without the need for clonal expansion, thereby providing an important link between the innate and adaptive immune systems [1].

T cell development occurs in the thymus and can be followed by the differential expression of the co-receptors CD4 and CD8. The earliest T cell progenitors are CD4/CD8 double negative (DN). The lineage choice between the αβ and γδ T cell lineages has already occurred by the late DN stage, where cells that have committed to the αβ lineage will continue to become CD4/CD8 double positive (DP), express the αβ TCR on the surface and eventually become either CD4 or CD8 single positive (SP). Unlike αβ T cells, development of γδ T cells remains poorly defined. Cells that are to become γδ T cells stay DN and start expressing the γδ TCR. The earliest γδ T cells are believed to be CD25^+^CD24^+^ [2]. Upon maturation γδ T cells down-regulate CD25 and up-regulate CD73 [3]. Later in their maturation, γδ T cells down-regulate CD24 to become fully mature CD24^−^CD73^+^ γδ T cells. Distinct γδ T cell subsets develop in waves and can be characterized by their expression of different Vγ segments in their TCR. Starting from around day 13 of gestation, the earliest γδ T cells appear in the thymus and express the Vγ3 chain (Vγ3^+^; Garman nomenclature [4]). These are followed by a wave of Vγ4^+^ γδ T cells. The last γδ T cells to develop are the Vγ1.1^+^ and Vγ2^+^ subsets, which are the two primary subsets in the adult thymus. Unlike conventional αβ T cells, γδ T cells are pre-programmed for their effector function during their development in the thymus [5, 6]. The major γδ effector T cells are IFNγ or IL-17 producers. They can be distinguished by their surface expression of CD27 and CD45RB with IFNγ producers being CD27^+^CD45RB^+^ and IL-17 producers being CD27^−^CD45RB^−^ [2, 6]. A recent study show that IL-17-producing γδ T cells develop primarily during the fetal and neonatal stages of life, while IFNγ-producing γδ T cells develop throughout all stages of life [7]. The different Vγ subsets are enriched within certain effector populations with Vγ1.1^+^ and Vγ3^+^ γδ T cells being primarily IFNγ producers and Vγ2^+^ and Vγ4^+^ γδ T cells being primarily IL-17 producers. The mechanisms behind the differential pre-programming of γδ T cells are still largely unknown. Some studies suggest that the pre-programming is determined by either the presence or absence of a selecting ligand within the thymus [5, 6], while others point toward cytokines and other local environmental factors as playing a role [8-12].

Inducible T cell co-stimulator (ICOS) is an inducible surface receptor of the CD28 co-receptor family. Unlike CD28 and CTLA4, ICOS does not bind CD80/CD86 but instead has a single distinct ligand called the ICOS ligand (ICOS-L). ICOS shows similar signaling properties as CD28, and constitutive expression of ICOS has been found to rescue CD28 deficiency [13]. Although CD28 and ICOS both activate the PI3K/AKT signaling axis [14, 15], ICOS has been reported to induce PI3K-independent calcium flux, indicating that other auxiliary pathways may be induced by ICOS signaling compared to CD28 signaling [16]. Studies of ICOS knockout (ICOS^−/−^) mice and *sanroque* mice have shown that while the overall T cell populations are largely unaffected by lack or constitutive expression of ICOS [17], ICOS is important for the development and effector function of specific T cell subsets [18]. The most prominent phenotype of ICOS^−/−^ mice is their loss of follicular helper T (Tfh) cells that are needed for germinal center formation and B cell antibody isotype switching [19-21]. Additionally, ICOS^−/−^ mice show reduced Th1- and Th2 responses manifesting in an inability to control viral and worm infections. Likewise, the development of Th1- and Th2-mediated autoimmune diseases is reduced in ICOS^−/−^ mice [22-24]. ICOS has also been found to be critical for Th17 differentiation and function in both mice and humans [25].

While T cells have been reported to develop normally in the thymus of ICOS^−/−^ mice [17], ICOS together with CD28 have been shown to be important for the development of both thymic natural killer T (NKT) cells and the recently discovered natural Th17 (nTh17) cells [26, 27]. Furthermore, ICOS:ICOS-L interactions have been implicated in the development of human thymic natural Treg cells [28]. ICOS is expressed by γδ T cells [29] already in the thymus, but little is known regarding its function on these cells. Until now, no studies have investigated the effect of ICOS signaling in the thymic development and effector programming of γδ T cells. In this study, we characterize ICOS expression on developing T cells in the thymus. We identify expression of ICOS on a subpopulation of immature γδ T cells enriched for markers associated with IL-17 production. Treatment with ICOS specific antibodies drastically and selectively reduced the development of IL-17-producing γδ T cells in the fetal thymus. Finally, we show that ICOS^−/−^ mice show altered subset distributions within their γδ T cell population with a 40-50% increase in IL-17-producing Vγ2^+^ γδ T cells in multiple immune organs and the skin and exhibit an increased skin response to the contact allergen 2,4-dinitrofluorobenzene (DNFB).

## RESULTS

### ICOS is expressed by mature CD4 or CD8 SP thymocytes

**Figure 1:**
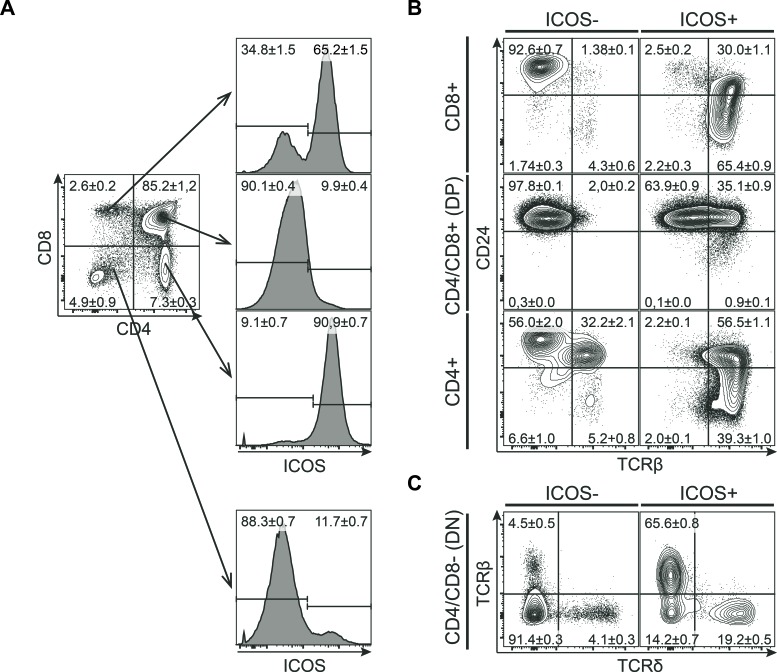
ICOS is expressed by mature CD4 or CD8 SP thymocytes Representative flow cytometric plots of thymocytes isolated from 7-8 weeks old mice and stained for CD4, CD8, CD24, ICOS, TCRβ and TCRδ. **A.** Expression of ICOS within the major thymic populations defined by CD4 and CD8. **B.** Expression of CD24 and TCRβ within the different ICOS^−^ (left) and ICOS^+^ (right) subpopulations. **C.** Expression of TCRβ and TCRδ within the ICOS^−^ (left) and ICOS^+^ (right) CD4/CD8 DN subpopulation. Numbers denote mean percentage &plusmn; standard error of mean (SEM) of the gated populations, (*n* = 12).

ICOS is nominally an inducible co-receptor but is also expressed at steady-state by several immune cell populations. To examine how ICOS is expressed during T cell development, we isolated thymocytes from C57BL/6 mice and analyzed expression of ICOS by flow cytometry. We found that ICOS is expressed by several populations of thymocytes in adult mice (Figure 1A). Almost all CD4 SP and more than 50% of the CD8 SP T cells express high levels of ICOS, whereas CD4/CD8 DP cells do not (Figure 1A).

During conventional αβ T cell development, progenitors start expressing the TCR at the CD4/CD8 DP stage at which point TCR selection occurs. After selection the remaining cells continue maturation and down-regulate CD24 before being exported from the thymus. To determine the relative timing of ICOS expression in developing T cells, we further characterized the expression of TCRβ and CD24 within the CD4/CD8 populations (Figure 1B). Within the DP, CD8 and CD4 SP populations ICOS is mainly expressed on TCRβ^+^ cells whereas the TCRβ^−^ cells are mainly ICOS^−^. Furthermore, the majority of ICOS^+^TCRβ^+^ cells had started to downregulate CD24 and showed high to intermediate expression of CD24. The CD8^+^CD24^high^TCRβ^−^ population most probably represented transitory immature SP cells (ISP) and they did not express ICOS. Taken together, these observations suggested that ICOS is expressed immediately after surface expression of the αβ TCR. Such an expression pattern is in good concordance with ICOS expression being induced by TCR signaling [30]. The CD4/CD8 DN cells contained a small population of cells expressing ICOS (Figure 1A). To determine which cell types within the DN fraction are expressing ICOS, we next analyzed the expression of TCRβ and TCRδ within the ICOS^+^ and ICOS^−^ DN cells (Figure 1C). The ICOS^+^ DN cells are highly enriched for cells expressing either the αβ or γδ TCR as compared with the ICOS^−^ cells (Figure 1C). This indicates that in the DN fraction, ICOS is primarily expressed on mature TCR-expressing cells similar to what we observed for the CD4 and CD8 SP populations (Figure 1B).

### ICOS is expressed by a large subset of immature γδ T cells enriched for Vγ2^+^CD45RB^low^ cells

**Figure 2:**
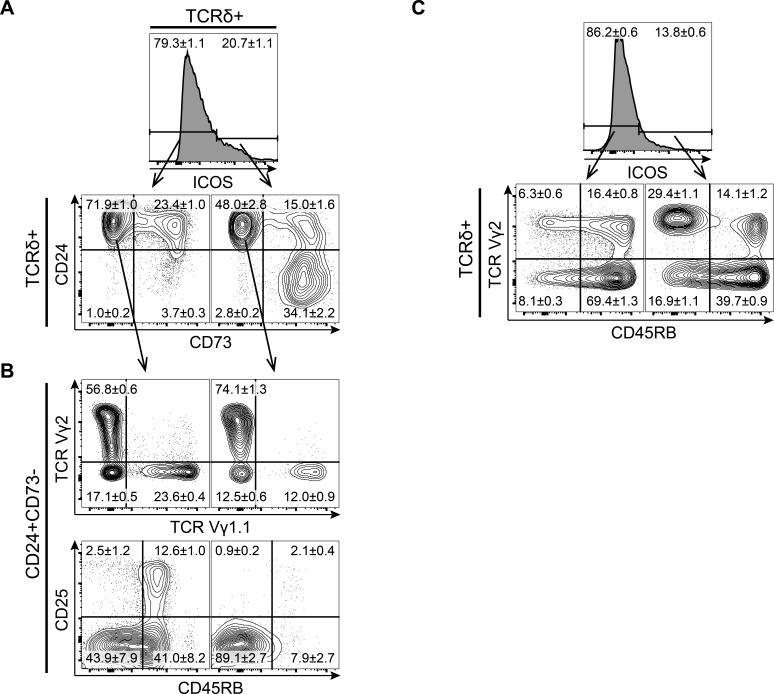
ICOS is expressed by a large subset of immature γδ T cells enriched for Vγ2^+^ CD45RB- cells Representative flow cytometric plots of CD4/CD8 depleted thymocytes or inguinal lymph nodes lymphocytes isolated from 7-8 weeks old mice and stained for ICOS, TCRδ, TCRβ, TCR Vγ2, TCR Vγ1.1, CD24, CD25, CD45RB and CD73. **A.** Expression of the maturation markers CD24 and CD73 on ICOS^−^ (left) and ICOS^+^ (right) cells within CD4/CD8 DN γδ T cells. **B.** Expression of TCR Vγ1.1, TCR Vγ2, CD25 and CD45RB within ICOS^−^ (left) and ICOS^+^ (right) immature CD24^+^CD73^−^ γδ T cells, (*n* = 8). **C.** Expression of TCR Vγ2 and CD45RB within ICOS^−^ (left) and ICOS^+^ (right) γδ T cells from inguinal lymph nodes, (*n* = 6). Numbers denote mean percentage &plusmn; SEM of the gated populations.

Developing γδ T cells up-regulate the TCR-inducible molecule CD73 during their early development and can be divided into three maturation stages by their expression of CD24 and CD73 with immature cells being CD24^high^CD73^−^, committed cells being CD24^high^CD73^+^ and mature cells being CD24^−^CD73^+^ cells [3]. If γδ T cells showed a similar expression pattern of ICOS as conventional αβ T cells, we would expect ICOS to be expressed on CD24^high^CD73^+^ cells as these cells are likely to have undergone recent TCR-mediated selection. Surprisingly, we found that ICOS was expressed by γδ T cells across all maturation stages including the immature CD24^high^CD73^−^ stage (Figure 2A). The expression of ICOS within the CD73^−^ population was surprising as both ICOS and CD73 are believed to be induced by TCR signaling. Therefore, we wanted to determine if ICOS expression on these immature cells marked a particular subpopulation of γδ T cells. In adult mice, γδ T cells can be divided into two major subsets by their expression of the Vγ1.1 or Vγ2 TCR chain. While CD27 has been shown to distinguish IFNγ (CD27^+^) and IL-17 (CD27^−^) producing γδ T cells [2], we found that all immature CD24^high^CD73^−^ cells express CD27, thus rendering CD27 a poor marker to distinguish IFNγ and IL-17-producing γδ T cells at this stage (data not shown). However, a recent study has associated low expression of CD45RB with IL-17 production in γδ T cells [6]. We found that immature CD24^high^CD73^−^ γδ T cells that express ICOS are enriched for Vγ2^+^ cells and almost exclusively express low levels of CD45RB (Figure 2B). We found a similar enrichment for Vγ2^+^CD45RB^low^ cells in the ICOS^+^ fraction of γδ T cells in the lymph nodes (Figure 2C). Finally, it is worth noting that the ICOS^+^ fraction does not contain many of the very early CD25^+^ γδ T cells, indicating that ICOS is up-regulated on immature γδ T cells after expression of the γδ TCR (Figure 2B).

Together, this showed that ICOS is expressed on a variety of different thymocyte populations in both the αβ and γδ T cell lineages. Unlike αβ T cells, which express ICOS during early post-selection stages, γδ T cells show ICOS expression across all maturity stages with a bias towards a subset of cells with IL-17-producing characteristics.

### Treatment with ICOS specific antibodies severely reduces the development of IL-17-producing γδ T cells

**Figure 3:**
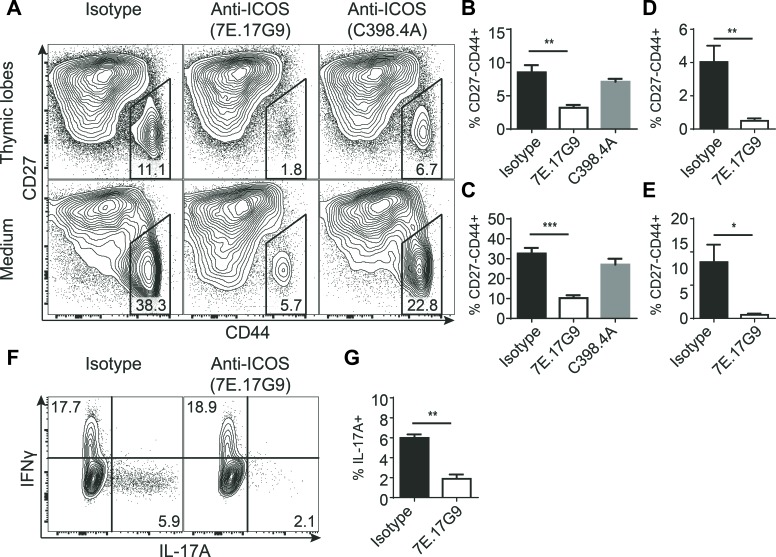
Treatment with ICOS specific antibodies severely reduces the development of IL-17-producing γδ T cells FTOC treated with anti-ICOS antibodies and cultured for 7 or 11 days. **A.** Representative flow cytometric plots showing CD27 and CD44 expression within γδ T cells harvested from the lobes (top) or culture medium (bottom) of e15 + 7 days FTOC. **B.-E.** Quantification of the CD44^+^CD27^−^ population percentages within γδ T cells harvested from thymic lobes (B, D) or culture medium (C, E) from e15 + 7 days FTOC (B, C) or e14 + 11 days FTOC (D, E) (*n* = 4). **F.** Representative flow cytometric plots showing intracellular IL-17A and IFNγ expression in γδ T cells from e14 + 11 days FTOC thymic lobes stimulated for 4 hours with PMA and ionomycin. **G.** Quantification of the IL-17A^+^ fraction of e14 + 11 days FTOC γδ T cells stimulated for 4 hours with PMA and ionomycin (*n* = 3). Each sample was pooled from a well containing 4-5 individual lobes. Bars denote mean percentage &plusmn; SEM of the gated populations.

The previous results indicated that ICOS might play a role in the development of IL-17-producing γδ T cells. Murine γδ T cells programmed to produce IL-17A develop mainly during fetal and neonatal life [7] and may be distinguished by their high expression of CD44 and lack of CD27 [2]. To investigate the role of ICOS interactions during the development and programming of γδ T cells, we made fetal thymic organ cultures (FTOC) with randomized thymic lobes from embryonic day 15 (e15) fetuses and treated them with antibodies added to the culture medium. Two different anti-ICOS antibodies were used, namely 7E.17G9 reported to be blocking [30-32] and C398.4A reported to be stimulating [33] as well as an isotype control. After 7 days in culture, the lobes and culture medium were harvested and T cell development was analyzed using flow cytometry (Figure 3A). Treatment with ICOS specific antibodies did not seem to affect the development of conventional αβ T cells as DP and CD4/CD8 SP populations appeared largely normal across the different treatments (data not shown). In contrast, within the TCRδ^+^ populations we found a highly significant and specific depletion of CD27^−^CD44^+^ cells in the cultures treated with 7E.17G9 both within the lobes and the culture medium (Figure 3A-3C). Although not statistically significant, cultures treated with C398.4A likewise showed a reduction in the CD27^−^CD44^+^ population (Figure 3A-3C). While the depletion of the CD27^−^CD44^+^ was highly significant, complete depletion was not obtained with neither antibody. As some γδ T cells are present before e15, we speculated that some cells evaded the anti-ICOS antibody treatment at a time point critical for the depletion. To determine if the incomplete depletion was due to the timing of the cultures, we conducted new 7E.17G9 antibody-treated FTOC experiments with randomized thymic lobes from embryonic day 14 (e14) fetuses. Similar to the e15 cultures, we observed a significant reduction in the CD27^−^CD44^+^ population in the e14 cultures treated with the 7E.17G9 antibody as compared with the isotype control (Figure 3D, 3E). We also stimulated thymocytes from the e14 cultured lobes with PMA and ionomycin for 4 hours and analyzed them for the expression of intracellular IL-17A and IFNγ. In concordance with the reduced CD27^−^CD44^+^ population, we found a significant decrease in the fraction of γδ T cells producing IL-17A in cultures treated with the 7E.17G9 anti-ICOS antibody (Figure 3F, 3G).

Taken together, these experiments show that treatment of FTOC with the ICOS specific antibodies selectively reduces development of IL-17-producing γδ T cells.

### Genetic ICOS deficiency causes subtle variations in fetal γδ T cell effector pre-programming

**Figure 4:**
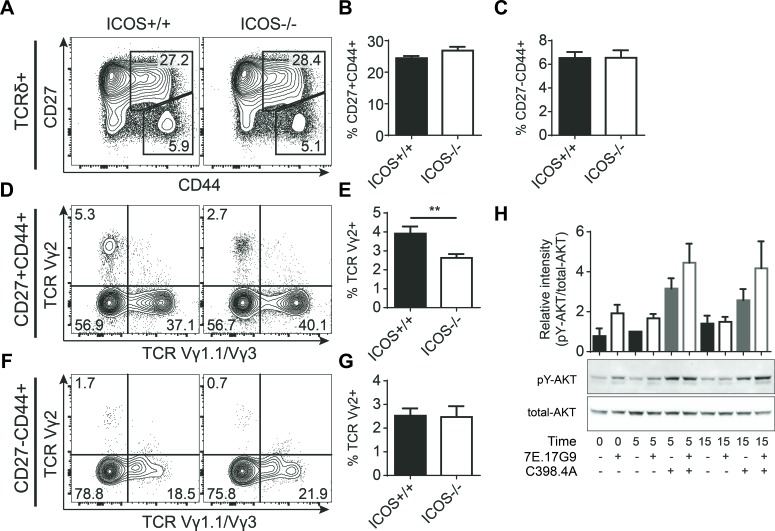
Genetic ICOS deficiency causes subtle variations in fetal γδ T cell effector pre-programming **A.-G.** FTOC of lobes harvested at embryonic day 15 from timed pregnant ICOS^+/+^ or ICOS^−/−^ mice and cultured for 7 days. A-C) Representative flow cytometric plots (A) and quantifications (B, C) showing expression of CD27 and CD44 within the γδ T cell population of cultured lobes. D-G) Expression of TCR Vγ1.1 and TCR Vγ2 within CD27^+^CD44^+^ (D, E) and CD27^−^CD44^+^ (F, G) γδ T cell populations. Thymic lobes were analyzed individually (*n* = 32). Bars denote mean percentage &plusmn; SEM of the gated populations. **H.** Western blot analysis of AKT phosphorylation in anti-CD3 propagated splenic T cells treated with anti-ICOS antibodies for 0, 5 and 15 minutes. Bars represent mean &plusmn; SEM of relative AKT phosphorylation (pY-AKT/total-AKT intensity) normalized to the 5 minute sample without anti-ICOS antibodies (*n* = 3).

The significant depletion of IL-17-producing γδ T cells in FTOC treated with the supposedly blocking 7E.17G9 anti-ICOS antibody encouraged us to determine if ICOS is required for the normal development of this subset of γδ T cells. To investigate this, we setup timed mating with pairs of mice genetically deficient for ICOS (ICOS^−/−^) or control mice (ICOS^+/+^). Similar to previous experiments, the fetuses were removed at embryonic day 15 (e15) and lobes were cultured in FTOC for 7 days followed by analysis using flow cytometry. Overall we saw very little difference between the ICOS^−/−^ and ICOS^+/+^ fetal thymic lobes. αβ T cell development (data not shown) as well as γδ T cell proportions, Vγ1.1, Vγ2 and Vγ3 subset distributions and surprisingly CD27^−^CD44^+^ and CD27^+^CD44^+^ populations were all unchanged (Figure 4A-4C). We did see a reduction of Vγ2^+^ cells within the otherwise unchanged CD27^+^CD44^+^ population (Figure 4D, 4E). This could indicate that while genetic ICOS deficiency does not reduce the development of fetal IL-17-producing γδ T cells (Figure 4F, 4G), it may subtly change Vγ2^+^ γδ T cell effector programming. The normal proportion of IL-17-producing γδ T cells (CD27^−^CD44^+^) in the ICOS^−/−^ fetal thymic lobes was in stark contrast to the phenotype seen in FTOC cultures treated with the 7E.17G9 anti-ICOS antibody. This made us speculate whether the 7E.17G9 antibody does in fact block ICOS signaling or if it shows ICOS-independent effects or agonistic properties. To test for ICOS-independent effects, we repeated similar FTOC experiments with ICOS^−/−^ fetal thymic lobes and treated the lobes with the 7E.17G9 anti-ICOS antibody or the isotype control. In these experiments we saw no effect of the 7E.17G9 antibody on the CD27^−^CD44^+^ population (data not shown), indicating that the observed antibody effect in ICOS^+/+^ FTOC is indeed ICOS dependent. In order to investigate if the 7E.17G9 antibody does in fact block ICOS signaling, we needed a larger number of ICOS expressing cells than we could get from γδ T cell populations. We therefore stimulated splenic T cells overnight with immobilized anti-CD3 antibodies. This induced ICOS expression on almost all the T cells (data not shown). These cells were then pre-incubated with high concentrations of the 7E.17G9 antibody for 5 minutes followed by addition of the stimulating C398.4A anti-ICOS antibody. Cells were harvested just prior to the addition of the C398.4A antibody (*t* = 0), after 5 (*t* = 5) and 15 minutes (*t* = 15), and ICOS stimulation was assessed by AKT phosphorylation using western blotting (Figure 4H). We found that both 7E.17G9 and C398.4A increased AKT phosphorylation as compared with untreated controls. Interestingly, pre-incubation with the supposedly blocking 7E.17G9 antibody did not reduce but rather increased AKT phosphorylation after subsequent addition of the stimulating C398.5A antibody at both the 5 and 15 minute time points.

Together, these observations indicated that the 7E.17G9 antibody exhibits ICOS dependent signaling properties and that it is not strictly a blocking antibody in our culture conditions. Consequently, these results indicate that the negative effect on the development of IL-17-producing γδ T cells induced by ICOS antibody treatment is mediated by agonistic ICOS signaling. Such negative regulation would not be present in ICOS^−/−^ thymic lobes and is further supported by the normal development of this subset in ICOS^−/−^ fetal thymic lobes.

### ICOS^−/−^ mice have an increased population of IL-17-producing Vγ2^+^ γδ T cells

**Figure 5:**
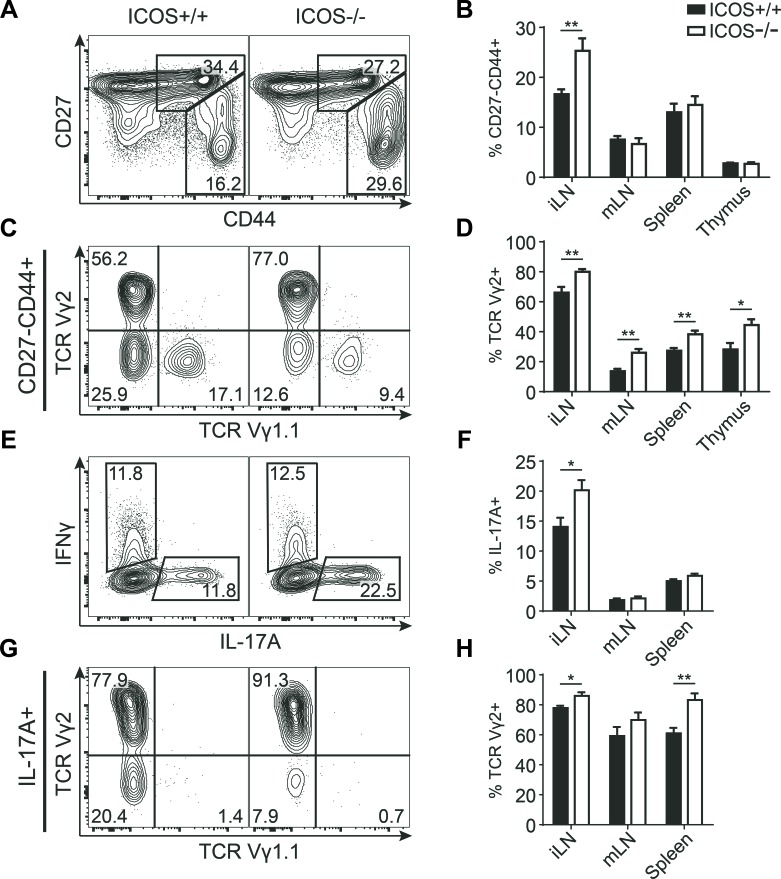
ICOS^−/−^ mice have an increased population of IL-17-producing Vγ2^+^ γδ T cells Comparison of γδ T cell populations from ICOS^+/+^ and ICOS^−/−^ mice. **A.** Representative flow cytometric plots showing CD27 and CD44 expression within γδ T cells from iLN. **B.** Quantification of the CD27^−^CD44^+^ fraction of γδ T cells from the indicated organs. **C.** Representative flow cytometric plots showing TCR Vγ1.1 and TCR Vγ2 expression within CD27^−^CD44^+^ γδ T cells from iLN. **D.** Quantification of the TCR Vγ2^+^ fraction of the CD27^−^CD44^+^ γδ T cell population from the indicated organs. **E.** Representative flow cytometric plots showing IL-17A and IFNγ expression in γδ T cells from iLN stimulated with PMA and ionomycin. **F.** Quantification of the IL-17A^+^ fraction of the γδ T cell population from the indicated organs stimulated with PMA and ionomycin. **G.** Representative flow cytometric plots showing TCR Vγ1.1 and TCR Vγ2 expression in IL-17A^+^ γδ T cells from iLN. **H.** Quantification of the TCR Vγ2^+^ fraction of IL-17A^+^ γδ T cells from the indicated organs. Bars denote mean percentage &plusmn; SEM of the gated populations (*n* = 8).

To determine how endogenous ICOS signaling affects the development of IL-17-producing γδ T cells as well as other lymphocyte populations, we performed a thorough T cell phenotyping of the thymus and secondary lymphoid organs in adult ICOS^−/−^ mice and compared with age-matched ICOS^+/+^ controls. Overall we found only few differences in the distribution of the major lymphocyte subsets between ICOS^−/−^ and ICOS^+/+^ mice (Supplementary Figure S1A-G) aside from the previously reported decrease in memory CD4^+^ T cells and a skewing of the B to T cell distribution in the lymph nodes [34, 35]. The inguinal lymph nodes (iLN) seemed to be most affected by the lack of ICOS (Supplementary Figure S1A). We found a selective and significant 40-50% increase in the CD27^−^CD44^+^ γδ T cell population in the iLN of ICOS^−/−^ mice (Figure 5A, 5B). This population also showed a significant enrichment of Vγ2^+^ cells, likely accounting for the overall increase in the population (Figure 5C, 5D). Interestingly, we found an even more drastic enrichment of Vγ2^+^ cells within the CD27^−^CD44^+^ γδ T cells of the other investigated immune organs (Figure 5D). The increase in Vγ2^+^ cells was not limited to immune organs as we also found a drastic increase in this population within the skin of ICOS^−/−^ mice (Supplementary Figure S1H, I). To verify that the increase in the CD27^−^CD44^+^ γδ T cell population in ICOS^−/−^ mice actually corresponded to an increase in IL-17-producing γδ T cells, we stimulated cells from the lymphoid organs with PMA and ionomycin and analyzed for intracellular cytokines by flow cytometry. We found a significant 40-50% increase in IL-17A^+^ γδ T cells in the iLN of ICOS^−/−^ mice similar to the increase in the CD27^−^CD44^+^ population (Figure 5E, 5F). It is worth noting that the IFNγ^+^ population is unchanged, showing that the increase in IL-17A^+^ γδ T cells is specific and not due to an overall increase in γδ T cell cytokine production (Figure 5E and Supplementary Figure S1E). Interestingly, the increase in IL-17A^+^ cells was specific to the γδ T cell lineage as the overall IL-17A^+^ lymphocyte population in the iLN and other organs seemed to be slightly albeit not significantly decreased (Supplementary Figure S1F, G). Similar to the CD27^−^CD44^+^ population we also found a significant enrichment of Vγ2^+^ cells within the IL-17A^+^ population indicating that the additional Vγ2^+^ cells are capable of IL-17 production (Figure 5G, 5H).

Together, these results showed that ICOS deficiency results in a significantly increased population of Vγ2^+^ γδ T cells in all investigated organs and that this enrichment increases the total size of the IL-17-producing γδ T cell population in the iLN.

### ICOS^−/−^ mice exhibit exacerbated responses during sensitization with DNFB

**Figure 6:**
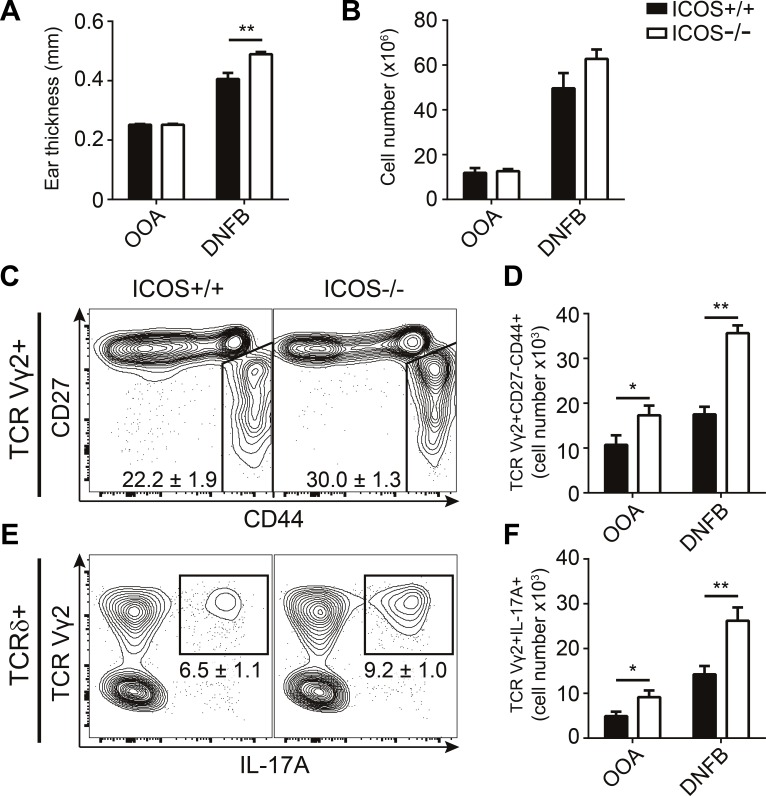
ICOS^−/−^ mice exhibit exacerbated responses during sensitization with DNFB Comparison of sensitization response to DNFB in ICOS^+/+^ and ICOS^−/−^ mice. **A.** Ear swelling measurements at day 5 after three consecutive days (day 0-2) treatments with DNFB or OOA (vehicle control). **B.** Quantification of lymphocyte numbers in the draining lymph nodes (dLN) of treated mice at day 5. **C.** Representative flow cytometric plots from DNFB treated mice showing CD27 and CD44 expression within TCR Vγ2^+^ γδ T cells from the dLN. **D.** Quantification of the number of TCR Vγ2^+^CD27^−^CD44^+^ γδ T cells. **E.** Representative flow cytometric plots from DNFB treated mice showing IL-17A and TCR Vγ2 expression in γδ T cells from dLN stimulated with PMA and ionomycin. **F.** Quantification of the number of TCR Vγ2^+^IL-17A^+^ γδ T cells in dLN after re-stimulation with PMA and ionomycin. Bars and numbers denote mean &plusmn; SEM from two independent experiments (*n* = 6).

Contact allergy is a T cell-mediated inflammatory disease manifesting as redness, swelling and itching of the skin following exposure to allergens. Both γδ T cells and IL-17 are required in the development of contact allergy [36, 37]. To investigate whether the increased numbers of IL-17-producing γδ T cells impact immune responses in ICOS^−/−^ mice, we sensitized ICOS^−/−^ and ICOS^+/+^ mice by painting the dorsal side of their ears with DNFB or vehicle control (olive oil in acetone; OOA) for three consecutive days. We measured the resulting acute ear swelling response 5 days after the first painting. DNFB treatment induced drastic ear swelling as compared with OOA treatment (Figure 6A). Importantly, ICOS^−/−^ mice showed significantly stronger ear swelling than their ICOS^+/+^ counterparts (Figure 6A). In parallel, we observed increased numbers of lymphocytes in the draining lymph nodes of ICOS^−/−^ compared to ICOS^+/+^ mice (Figure 6B). To determine if the exacerbated sensitization response in ICOS^−/−^ was accompanied by increased IL-17A-producing Vγ2^+^ γδ T cells, we analyzed the draining lymph nodes cells by flow cytometry. We found a significant increase in both the number and fraction of CD27^−^CD44^+^ and IL-17A^+^Vγ2^+^ γδ T cells in ICOS^−/−^ mice compared to ICOS^+/+^ mice (Figure 6C-6F). Taken together, these observations support that the increased numbers of IL-17A-producing γδ T cells in ICOS^−/−^ mice play a physiologically relevant role during immune responses.

## DISCUSSION

In this study, we have characterized ICOS expression in developing T cells in the thymus. We found ICOS expression on αβ T cells that have recently received signals through their TCR. However, we also found expression of ICOS on a subpopulation of immature γδ T cells thought not to have received TCR signals. This population was highly enriched for Vγ2^+^ cells expressing low levels of CD45RB, both of which are associated with IL-17A-producing γδ T cells in the periphery. By anti-ICOS antibody treatment we were able to drastically and selectively reduce the development of IL-17-producing γδ T cells in the fetal thymus. As similar cultures with ICOS^−/−^ thymic lobes did not show an equivalent reduction, we could conclude that the impaired development of IL-17-producing γδ T cells caused by the anti-ICOS antibodies in ICOS^+/+^ mice was directly mediated by ICOS signaling. Supporting the notion that ICOS signaling may oppose IL-17-producing γδ T cell development, we found a specific enrichment of IL-17-producing Vγ2^+^ γδ T cells in multiple immune organs and the skin of ICOS^−/−^ mice. These observations indicated that while ICOS expression is dispensable for IL-17-producing γδ T cell development, ICOS plays a role in modulating the programming of these cells, particularly the ones utilizing the Vγ2 segment. While anti-ICOS antibody-mediated reduction of these cells was clearly caused during development, we could not determine whether the increase in IL-17-producing γδ T cells in ICOS^−/−^ mice is due to increased thymic development or homeostatic changes in the periphery. However, the facts that ICOS is expressed at a very early stage in Vγ2^+^ γδ T cells from ICOS^+/+^ mice and that the specific enrichment of IL-17-producing Vγ2^+^ γδ T cells is present already in the thymus of ICOS^−/−^ mice suggest that changes in thymic development and programming account for at least a part of the phenotype.

It is intriguing that the 7E.17G9 antibody, reported to be blocking [30-32], exerted agonistic ICOS signaling in our experiments (Figure 4H). Agonistic signaling induced by 7E.17G9 was further supported by the apparent discrepancy between antibody treated FTOC and the comparable ICOS^−/−^ FTOC experiments (Figure 3 and Figure 4A-4G). Furthermore, adult ICOS^−/−^ mice show an increase in the IL-17-producing γδ T cell population in stark contrast to the drastic depletion seen in 7E.17G9 antibody treated thymic lobes (Figure 3 and Figure 5). While the C398.4A antibody induced high levels of AKT phosphorylation, its effect on γδ T cell development was more modest. This suggests that these antibody clones induce different signals downstream of ICOS. This is supported by the existence of auxiliary ICOS signaling pathways and that the investigated clones have been found to bind distinct epitopes [16, 30, 38].

**Figure 7:**
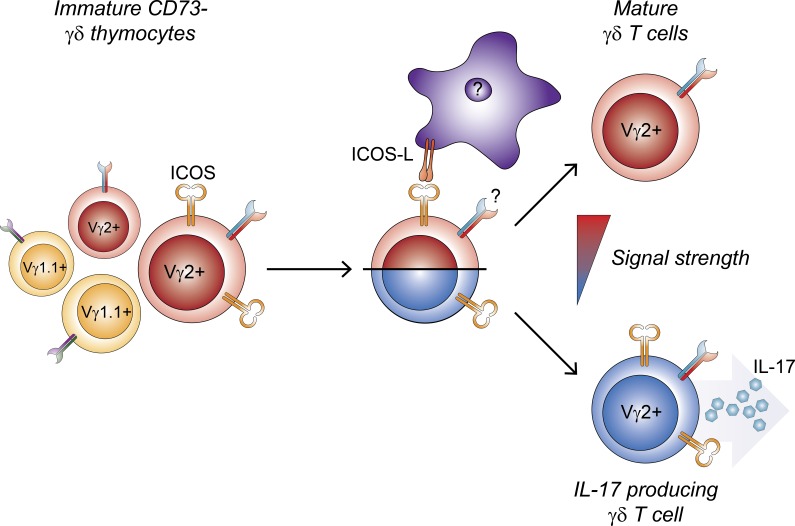
Development of interleukin-17-producing Vγ2^+^ γδ T cells is reduced by ICOS signaling in the thymus ICOS is expressed by a subpopulation of immature Vγ2+ thymocytes which are biased to develop toward an IL-17 producing phenotype. Signaling through ICOS increases the integrated signal strength above the threshold for IL-17 pre-programming, thus diverting developing γδ thymocytes away from this fate.

Effector pre-programming of γδ T cells as well as commitment to the γδ lineage have been closely tied to the TCR signals received by the γδ T cells during their development [39-42]. It has previously been reported that CD73 is expressed after TCR-ligand interaction, and that CD73 marks γδ T cells that have committed to the γδ T cell lineage [3]. This makes the appearance of ICOS, another TCR-inducible molecule, on CD73^−^ γδ T cells puzzling. IL-17-producing γδ T cells have been proposed to be programmed by the lack of or weak TCR-ligand interactions [5, 6], whereas strong TCR-ligand interactions induce programming of IFNγ-producing γδ T cells or IFNγ- and IL-4-producing γδ NKT cells. This could explain a possible mechanism by which co-stimulatory ICOS signaling drives the integrated signal strength above the upper limit for IL-17 imprinting, thereby programming IL-17-producing γδ T cell progenitors away from their normal fate (Figure 7). In addition to the rapid innate-like producers of IL-17 or IFNγ described above, recent evidence accumulates for the existence of an adaptive-like subset of γδ T cells [43-45]. Similar to the results of other studies identifying factors required for development of IL-17-producing γδ T cells [8, 9, 11, 12], we do not observe a reciprocal change in IFNγ-producing γδ T cells (Figure 3F, Figure 5E-5F and Supplementary Figure S1E). This suggests that IL-17 and IFNγ-producing γδ T cells are not necessarily in direct competition, so that reduction of one subset leads to the increase in the other. We suggest that, while ICOS signaling drives a supra-threshold response for IL-17-producing γδ T cell differentiation, the γδTCR of the CD73^−^ICOS^+^ cells are unlikely to encounter high-affinity ligand as this would already have driven the cells away from the IL-17-producing fate. Thus, they do not receive sufficiently strong signals required for IFNγ pre-programming and may instead be driven toward a different fate, possibly the recently identified adaptive-like subset.

While the role of ICOS has been thoroughly investigated in αβ T cells, this is one of the first studies showing a role of ICOS in γδ T cells. A recent study has shown promising indications for utilizing the ICOS-ICOS-L pathway in the context of anti-CTLA-4 immunotherapy for cancer treatment [46]. Our study highlights the need for a deeper knowledge of the different cell types affected by the ICOS-ICOS-L pathway to determine the utilities and pitfalls such treatments would include. This concern is further highlighted by ICOS^−/−^ mice showing exacerbated skin response to DNFB, despite having reduced co-stimulatory capacity and reduced Th17 development. While ICOS has been shown to be important for the differentiation of human Th17 cells, it remains to be determined if human IL-17-producing γδ T cells are similarly or oppositely regulated by ICOS as in mice, and how this affects potential therapies.

## MATERIALS AND METHODS

### Mice

ICOS^−/−^ mice (B6129P2-Icos*^tm1Mak^*/J) were purchased from The Jackson Laboratory. For FTOC, timed pregnant C57BL/6 were obtained from Taconic (Ry, Denmark) or bred together with ICOS^−/−^ mice in specific pathogen-free facilities at the Department of Experimental Medicine, Faculty of Health and Medical Sciences, University of Copenhagen, in accordance with national animal-protection guidelines (license no. 2012-15-2934-00663).

### Flow cytometry and cytokine staining

Antibodies against CD4 (RM4-5), CD8 (53-6.7), CD19 (1D3), CD24 (M1/69), CD25 (PC61), CD27 (LG.3A10), CD44 (IM7), CD45RB (C363-16A), CD62L (MEL-14), CD73 (TY/11.8), ICOS (C398.4A), TCRδ (GL-3), TCRβ (H57-597), TCRVγ1.1 (2.11), TCRVγ2 (UC3-10A6), TCRVγ3 (536) and NK1.1 (PK136) were purchased from BD Biosciences (Franklin Lakes, NJ, USA), BioLegend (San Diego, CA, USA) or eBiosciences (San Diego, CA, USA). Fixable Viability Dye (eFluor780 or eFluor506; eBiosciences, San Diego, CA, USA) or 7-AAD (BioLegend, San Diego, CA, USA) was used to exclude dead cells in the analysis.

Thymus, spleen and lymph nodes were harvested from 7-8 week old C57BL/6 or ICOS^−/−^ mice. For enrichment of thymic γδ T cells, thymocytes were depleted with anti-CD4 and anti-CD8 magnetic beads (Miltenyi Biotec). For all stainings, antibodies were diluted in Brilliant Stain Buffer (BD Biosciences, San Diego, CA, USA) and stained for 30-40 minutes in the dark on ice. For intracellular cytokine staining, single-cell suspensions were stimulated with PMA (25 ng/ml) and ionomycin (625 ng/ml) in complete medium including monensin (2.08 &micro;g/ml) for 4 hours at 37&deg;C. Cells were stained for surface markers, fixed and permeabilized using BD Cytofix/Cytoperm (BD Biosciences). Permeabilized cells were stained with anti-IL-17 (TC11-18H10) or anti-IFNγ (XMG1.2). All samples were analyzed on a BD LSRFortessa at the Core Facility for Flow Cytometry, Faculty of Health and Medical Sciences, University of Copenhagen. Data was analyzed using the FlowJo software (Treestar, Ashland, OR, USA).

### Fetal thymus organ culture and anti-ICOS treatment

Fetuses were harvested from timed-pregnant C57BL/6 or ICOS^−/−^ mice at day 14 or 15 of gestation (measured as days after female was plugged), after which thymic lobes were cultured on isopore membrane filters (Merck Millipore Ltd.) placed in the surface tension of 2 mL DMEM (20% heat-inactivated fetal bovine serum, 10 mM HEPES, 0.05 mM β-mercaptoethanol, 4 mM L-glutamine, 100 IU/mL penicillin and streptomycin) in 12 well plates (ThermoScientific). 4-8 lobes were cultured on each membrane filter. Half-way through cultures, the medium was carefully removed and replaced. After 7 or 11 days for e15 and e14 cultures, respectively, the lobes and culture medium was harvested and minced through a 70&micro;m cell-strainer followed by flow cytometric analysis as described above.

For anti-ICOS treated FTOC, LEAF-purified anti-ICOS (7E.17G9 and C398.4A) or anti-Rat IgG2b isotype was purchased from BioLegend (San Diego, CA, USA) and added to the culture medium to a final concentration of 5 &micro;g/mL.

### Anti-ICOS stimulation and quantification of AKT-phosphorylation by western blotting

Spleens were harvested from 7-8 week old C57BL/6 mice and minced through a 70 &micro;m cell strainer. After washing, the cells were resuspended in 0.165 M NH_4_Cl and left undisturbed for 11 minutes on ice to lyse red blood cells. Splenocytes were enriched for T cells by depletion of B cells using magnetic anti-mouse IgG beads (ThermoFisher; cat. no. 11031). To induce ICOS expression, cells were cultured in flat-bottom 96 well plates (ThermoScientific) pre-coated with 5 &micro;g/mL anti-CD3 (145-2C11) overnight. Cells were harvested and 5×10^6^ cells were aliquoted into tubes according to treatment and starved for 30 minutes in 100 &micro;l PBS (containing 10 mM HEPES) at 37&ordm;C. Cells were pre-incubated 5 minutes with anti-ICOS (7E.17G9) prior to addition of anti-CD3 (145-2C11) and anti-ICOS (C398.4A) all to a final concentration of 50 &micro;g/mL and kept in water bath at 37&ordm;C throughout. At 0, 5 and 15 minutes after addition of antibodies, 30&micro;l was harvested from each treatment and added to 900 &micro;l ice cold PBS and put on ice. Samples were lysed and analyzed by western blotting as previously described [47] using anti-Phospho-AKT (Thr308) Rabbit mAb and anti-AKT (Cell Signaling Technology)

### Skin sensitization to DNFB

Mice were painted with 25 &micro;l 0.15% 2,4-dinitrofluorobenzene (DNFB; Sigma-Aldrich) in 1:4 olive oil:acetone (OOA) mixture on the dorsal side of both ears for three consecutive days (days 0-2). On day 5, mice were euthanized and ear thickness was measured on both ears using an engineer's micrometer (Mitutoyo, Tokyo, Japan). Ear draining (d)LN were excised and analyzed by flow cytometry as described above.

### Statistical analysis

Statistical analyses were performed using Student's t test with a 5% significance level, un-paired observations and equal variance, (*p ≤ 0.05; **p ≤ 0.01; ***p ≤ 0.001).

## SUPPLEMENTARY MATERIAL FIGURE



## Abbreviations

DN: Double negative
DNFB: 24-dinitrofluorobenzene
DP: Double positive
FTOC: Fetal thymic organ culture
ICOS: Inducible co-stimulator
IFNγ: Interferon-γ
IL-17: Interleukin-17
LN: Lymph node
MHC: Major histocompatibility complex
OOA: Olive oil in acetone (1:4)
TCR: T cell receptor
