# Supplementary material for: Development of interleukin-17-producing Vγ2+ γδ T cells is reduced by ICOS signaling in the thymus
Source: Oncotarget. 2016 Mar 29;7(15):19341–54. doi: 10.18632/oncotarget.8464 (PMC4991387; doi:10.18632/oncotarget.8464)
Supplement: Supplementary file 1 [file oncotarget-07-19341-s001.pdf]

# Development of interleukin-17-producing Vγ2$^{+}$ γδ T cells is reduced by ICOS signaling in the thymus

## Supplementary Material

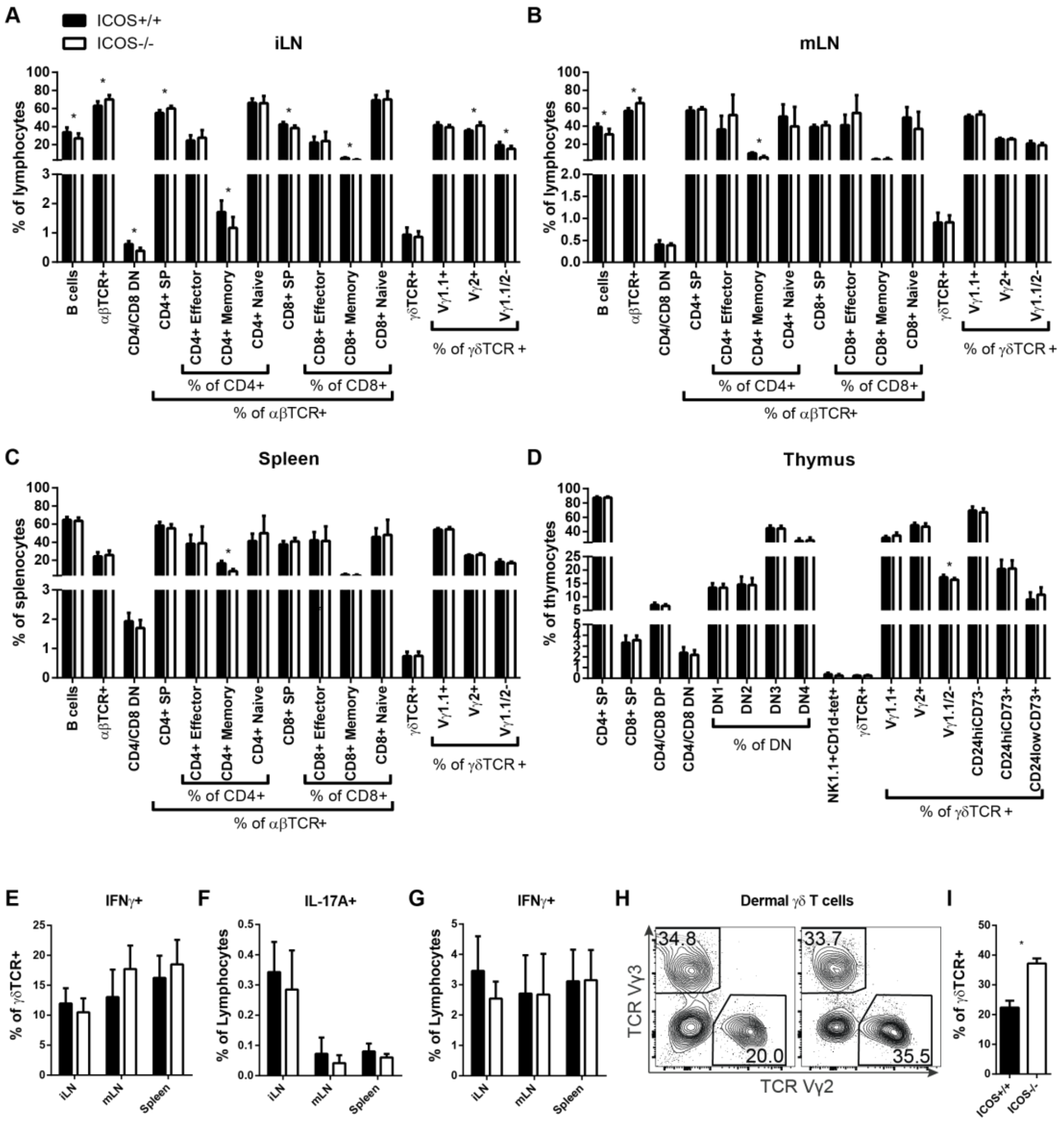


## Supplementary Figure S1: ICOS$^{-/-}$ T cell phenotype overview

**A-D)** Comparison of lymphocyte populations from **A)** inguinal lymph nodes (iLN), **B**) mesenteric lymph nodes (mLN), **C**) spleen and **D)** thymus of ICOS$^{+/+}$ and ICOS$^{-/-}$ mice. Effector, memory and naïve CD4$^{+}$ and CD8$^{+}$ cells are defined by being CD62L$^{-}$CD44$^{-}$, CD62L$^{-}$CD44$^{+}$ and CD62L$^{+}$CD44$^{-}$, respectively (n=8). **E)** Quantification of the IFNγ$^{+}$ fraction of the γδ T cell population from the indicated organs after stimulation with PMA and ionomycin. **F-G)** Quantification of the fraction of **F**) IL-17A$^{+}$ and **G**) IFNγ$^{+}$ T cells from the indicated organs after stimulation with PMA and ionomycin (n=8). **H)** Representative flow cytometric plots showing TCR Vγ2 and TCR Vγ3 expression in γδ T cells from the skin of ICOS$^{+/+}$ and ICOS$^{-/-}$ mice. **I)** Quantification of the TCR Vγ2$^{+}$ fraction of γδ T cells from the skin of ICOS$^{+/+}$ and ICOS$^{-/-}$ mice (n=2). Bars denote mean percentage ± SEM of the gated populations (*p ≤ 0.05).
